# Analysis of Conformational Motions and Residue Fluctuations for *Escherichia coli* Ribose-Binding Protein Revealed with Elastic Network Models

**DOI:** 10.3390/ijms140510552

**Published:** 2013-05-21

**Authors:** Hai Yan Li, Zan Xia Cao, Li Ling Zhao, Ji Hua Wang

**Affiliations:** 1 Shandong Provincial Key Laboratory of Functional Macromolecular Biophysics, Dezhou University, 566 University Road West, Dezhou 253023, China; E-Mails:dzxylhy@163.com (H.Y.L.); qiayilai@mail.ustc.edu.cn (Z.X.C.); zhaoll@dzu.edu.cn (L.L.Z.); 2 Department of Physics, Dezhou University, 566 University Road West, Dezhou 253023, China

**Keywords:** ribose-binding protein, elastic network model, conformational motions

## Abstract

The ribose-binding protein (RBP) is a sugar-binding bacterial periplasmic protein whose function is associated with a large allosteric conformational change from an open to a closed conformation upon binding to ribose. The open (ligand-free) and closed (ligand-bound) forms of RBP have been found. Here we investigate the conformational motions and residue fluctuations of the RBP by analyzing the modes of motion with two coarse-grained elastic network models, the Gaussian Network Model (GNM) and Anisotropic Network Model (ANM). The calculated B-factors in both the calculated models are in good agreement with the experimentally determined B-factors in X-ray crystal structures. The slowest mode analysis by GNM shows that both forms have the same motion hinge axes around residues Ser103, Gln235, Asp264 and the two domains of both structures have similar fluctuation range. The superposition of the first three dominant modes of ANM, consisting of the rotating, bending and twisting motions of the two forms, accounts for large rearrangement of domains from the ligand-free (open) to ligand-bound (closed) conformation and thus constitutes a critical component of the RBP’s functions. By analyzing cross-correlations between residue fluctuation and the difference-distance plot, it is revealed that the conformational change can be described as a rigid rotation of the two domains with respect to each other, whereas the internal structure of the two domains remains largely intact. The results directly indicate that the dominant dynamic characteristics of protein structures can be captured from their static native state using coarse-grained models.

## 1. Introduction

Many proteins have been documented as undergoing functionally important conformational changes in association with binding to their specific ligands. They are important in a variety of protein functions such as catalysis, regulation of activity, signaling, allosteric regulation, complex formation, and substrate binding [1,2]. Periplasmic binding proteins (PBPs) are bacterial receptors that exhibit dramatic conformational changes upon ligand binding. These proteins mediate a wide variety of fundamental processes including transport, chemotaxis, and quorum sensing [3–5]. Most PBPs participate in the transport of solute molecules into the cytoplasm via ABC transporters [4,6]. Their targets include critical nutrients such as carbohydrates, amino acids, vitamins, and ions. PBPs consist of two domains connected by a hinge region, with a ligand-binding site located at the interface between the two domains. Ligand-free PBPs adopt an open conformation in which the inter-domain interface is exposed to solvent. Solute binding induces a conformational change to form a closed state in which the ligand is bound at the domain interface and buried by the surrounding protein [7–9]. The resulting complex possesses a protein-binding surface not present in the open form; therefore, the complex can be recognized by membrane-bound receptors [10]. Periplasmic binding proteins (PBPs) provide a model system for investigating the conformational change upon binding to ligand and they have been extensively studied by crystallography [9,11,12], NMR [7,13,14], Molecular dynamics (MD) simulation [15–17], Monte Carlo method [18], elastic network theory [19,20] and other biophysical techniques [21–23].

**Figure 1:**
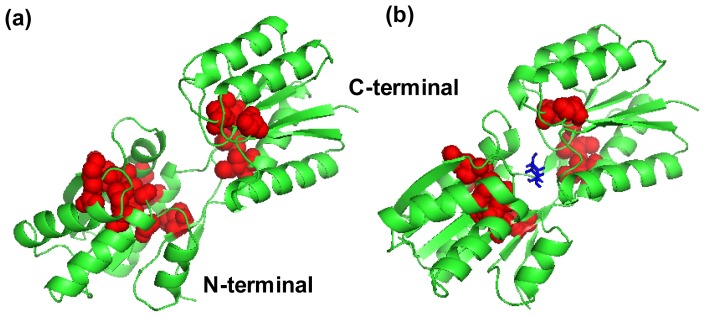
Ribose-binding protein (RBP) in (**a**) an open ligand-free conformation (PBD: 1URP) and (**b**) a closed ligand-bound conformation (PDB: 2DRI), the ribose is marked in blue. The peak residues are marked in red. All the peak residues form a contiguous cluster distributed on the surface of RBP facing the ribose-binding pocket.

The ribose-binding protein (RBP) from *Escherichia coli* is one of the representative structures of the PBPs. Binding of ribose at the cleft between two domains causes a conformational change corresponding to a closure of two domains around the ligand. The RBP has been crystallized in the open (PDB ID: 1URP [9]) and the closed conformation (PDB ID: 2DRI [11]) (Figure 1) and the structures differ by a 41.3° rotation of the *N*-terminal domain relative to the *C*-terminal domain calculated by Dyndom [24]. Like other members in the bacterial superfamily of PBPs, the ribose binding protein is an α/β protein that is composed of two highly similar globular domains. The C-terminal domain consists of two separate peptide segments, *i.e*., residues 1–100 and 236–259, which includes four α-helices and four β-strands. The N-terminal domain is comprised of residues 108–231 and 269–271, which contains five α-helices and four β-strands. The two domains are connected by three β-strands as a flexible hinge region (residues 101–107, 232–235 and 260–268), which permits the domains of the protein to rotate with respect to the other in response to ligand binding at the interface between the two domains.

RBP has been widely studied by experimental and theoretical methods. Vercillo *et al*. chose the RBP to investigate the effect of the large-scale site-directed mutation on protein stability [21]. Small-angle X-ray scattering experiments were carried out for the RBP and two related maltose-, glucose/galactose-binding proteins of Gram negative bacteria. All were shown to be monomers that decrease in radius of gyration on ligand binding [22]. Haspel *et al.* investigated the application of a robotics-inspired method, using backbone and limited side chain representation and a coarse grained energy function to trace large-scale conformational motions of RBP and other three proteins [25]. The opening/closing mechanism of the RBP was studied via umbrella sampling molecular dynamics, and a free energy landscape as a function of the hinge and twist angles was proposed [16]. MD simulation is an effective method to obtain the detailed microscopic dynamics of proteins, which has been widely used to study the dynamic processes of biological macromolecules [15–17]. A great deal of effort in recent years has been put into accelerating molecular dynamics techniques using, e.g., parallel tempering [26] or replica exchange [27]. However, MD simulation has some limitations to study the functional movements of a protein because it consumes a vast amount of computation resource. To reduce such a computational burden, Gaussian network model (GNM) [28–31] and Anisotropic network model (ANM) [29,32] have been shown to successfully capture the large-scale collective motions relevant to protein functions. ANM/GNM assumes an elastic network structure, formed by springs that connect the close neighboring *C_α_* atoms in the 3-dimensional structure of proteins. The method is coarse-grained by using a single site per residue and assuming that all the residues in a cutoff distance are in contact. Several previous studies have proved that the results of the GNM and ANM are in agreement with those of MD simulation [19,20,33]. In this work, we used GNM and ANM to analyze RBP’s conformational motions and residue fluctuations that have direct consequences on the transport of ribose, which lends support to the effectiveness of these coarse-grained models in the analysis of the structure-function relationship of proteins and their complexes.

## 2. Results and Discussions

### 2.1. Comparison of Equilibrium Fluctuations of GNM and ANM with Experimental Temperature Factors

To evaluate the availability of applying the GNM and ANM methods to study RBP, the B-factors are calculated with the two methods and then compared with the data from X-ray crystallography. The B-factors are related to the mean square fluctuations of individual residues according to Equation (4). The factor *k_B_T*/*γ* is essentially a force constant for the virtual springs connecting *C_α_* atoms and sets the overall scale factor. The resulting *k_B_T*/*γ* values in the GNM, chosen for each protein so as to scale overall the calculated curves to best fit the experimental data, are 5.64 and 8.71 Å^2^, respectively. The values calculated in the ANM are 2.128 and 3.161 Å^2^, respectively.

**Figure 2:**
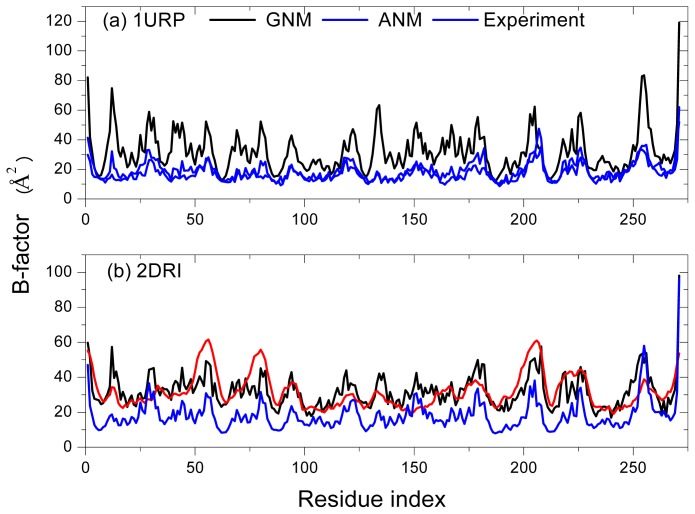
Comparison of theoretical and experimental B-factors for (**a**) 1URP and (**b**) 2DRI.

Figure 2a,b display the comparison between the calculated B-factor of *C_α_* atoms and the experimental data from X-ray crystallography of the open-unligand form (PDB code1URP) and the closed-ligand form (PDB code 2DRI), respectively. The correlation coefficient of the B-factor between the experiment and GNM is 0.583, 0.772 for 1URP and 2DRI, respectively. That between the experiment and ANM is 0.436, 0.794 for 1URP and 2DRI, respectively. The results are similar to those studies for other proteins [19,34]. The GNM and ANM can be used for further investigation of the conformational motions and fluctuations of ribose-binding protein. It is found that the closed-ligand structure gives a higher correlation coefficient than the open-unligand structure, which maybe reflects the effect of the ligand ribose on the residue fluctuations of the protein. Otherwise, the better correlation might be due to a better quality of the experimental data for closed-ligand structure (Resolution = 1.6 Å instead of 2.3 Å for the unbound form). We have calculated the correlation coefficient of the B-factor between the experiment and GNM of the five other proteins belonging to the *Escherichia coli* family and listed the data in Table 1. There is no obvious evidence to show that the closed-ligand structure can obtain a higher correlation coefficient than the open-unligand structure due to the effect of the ligand or a higher correlation coefficient is due to a better quality of the experimental data.

### 2.2. The Slow Mode of the Motion

**Figure 3:**
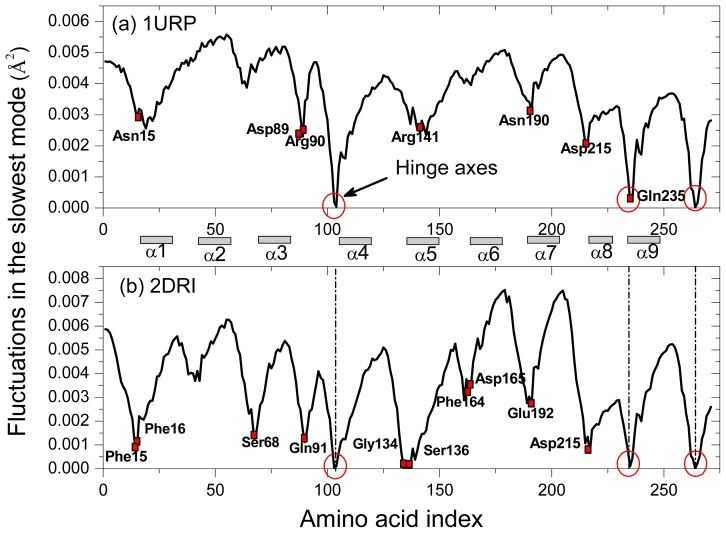
Comparison of the mean square fluctuations due to slowest mode for (**a**) 1URP and (**b**) 2DRI. The hinge axes are indicated by a red circle. The binding residues and the residues forming hydrogen bonds in the closed structure are marked by a red square.

**Figure 4:**
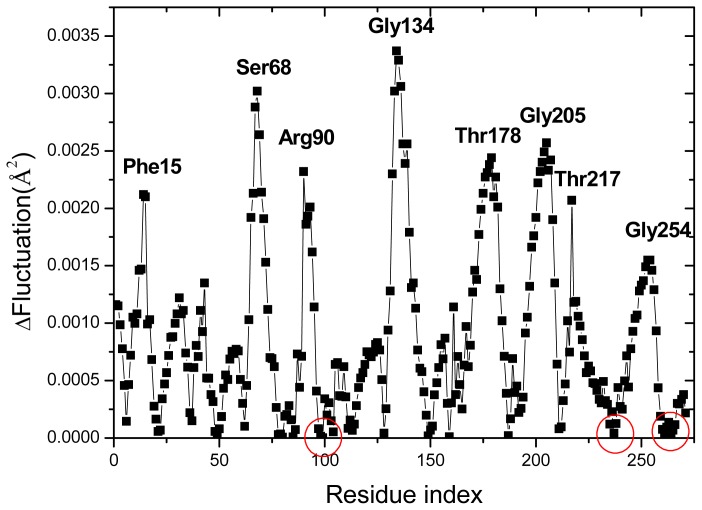
Calculated fluctuation difference between the open-apo and closed-ligand structures using the slowest mode of GNM.

The low-frequency, collective modes of motion are especially significant, since they are essential for the function of the protein [42]. Figure 3 shows the slowest mode of both structures calculated by the GNM, which corresponds to the motion mode with the minimum frequency. The difference fluctuations of the slowest mode between the two structures are shown in Figure 4. With the slowest mode from GNM, the two domains of the protein and the hinges axes between them are highly distinguished. The two structures have the common hinge axes located around Ser103, Gln235, Asp264, with the fluctuation values approximating to zero (the hinge axes are indicated by red circle in Figure 3). Around the hinge axis, the major domain movements occur. Some proteins belong to the *Escherichia coli* family such as glutamine-binding protein (GlnBP), the open-closed conformational transition mainly exhibits as the large movement of the small domain [19]. From Figure 3, the two domains for both structures of RBP have similar fluctuation range and this is due to the two domains have approximately the same structure and size. It indicates that the open-closed conformational transition of RBP is common controlled by the two domains.

In the closed conformation of RBP (2DRI), residues Asn13, Phe15, Phe16, Asp89, Arg90, Arg141, Asn190, Asp215 and Gln235 are known to be ligand-binding sites [16] and located in the jaws of the ligand docking, which are all located at minima in the curve displayed in Figure 3b. Comparing the slowest mode of the closed structures with that of the Open structure, it can be seen that the fluctuations of those residues in the closed structures are reduced remarkably, this is helpful for ligand release or binding. The fluctuation decrease of these residues implies that the binding pocket became more stable in the closed structure than in the open-apo structure. Previous study show that the conformation of the binding pocket in RBP undergoes little ligand-induced rearrangement [43]. However, in E. coli leucine-binding protein, not only the hinge region, but also loops and amino acid sidechains in the binding pocket show ligand-induced changes, many of which are restricted to one domain [35]. Although the rearrangement in the binding pocket of RBP is tiny, it can also be captured by ENM.

Three hydrophobic residues, Asp 215, Phe15, Phe16, have small fluctuation in the closed structure, which make hydrophobic stacking interactions with the ligand. Gln235, which is located in the second hinge segment, makes hydrogen bonds to both domains as well as to the ligand in the closed protein; it is the only hinge residue that interacts directly with sugar. This unique situation in RBP suggests that Gln235 will have a special role in the conformational change. Other minima are observed near residues Ser68, Gln91, Gly134 and Ser136. The four residues were reported to form the only three interdomain hydrogen bonds (between N-68 and O-134, between OG-68 and O-136, and between OG-136 and N-91) in the closed structure [16]. From Figure 4, it is found that the difference fluctuations of the residues Ser68, Gln91, Gly134 and Ser136 in the open-apo structure give a higher fluctuation than the closed-ligand structure, which reflects the effect of the interdomain hydrogen bonds on the residue fluctuations of the protein. It is therefore reasonable that these regions should alter their local structure when contact between the domains is lost. The residue Gly134 is located in a β-turn (residues 132–135) near the exterior edge of the ligand binding cleft and is associated with both the exposed surface in the ligand-bound RBP and interdomain contact, alteration of which might influence functional behavior [11]. The umbrella sampling molecular dynamics studies [16] indicate that the three new interdomain hydrogen bonds (between N-Asn12 and O-Asp165, between N-Asn13 and O-Phe164, between N-Asn13 and O-Glu192) are found in the closed ribose-free conformations on the opposite side of the ribose-binding site. It is noticeable that the five residues mention above also located at local minima fluctuation in the closed-ligand structure.

The highly dynamical nature is reflected not only in the large amplitude of domain fluctuations in the GNM global mode but also in the directions of domain motions as revealed by the ANM analyses. In the ANM, the fluctuations are no longer assumed to be isotropic, but their X-, Y-, and Z-components are evaluated separately. X-ray structures of similar binding proteins in their ligand-bound and ligand-free forms have shown that two initially separated domains close down around the ligand by virtue of an approximately 30° rotation in the hinge that connects them [44]. After this change, the binding proteins can interact effectively with specific transport complexes in the inner membrane and so direct the entry of ligand into the cell.

**Figure 5:**
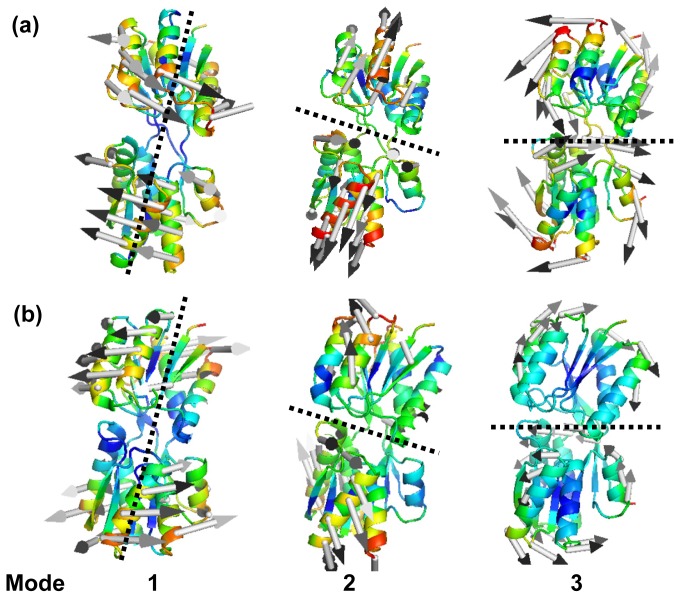
Mean-square fluctuations of the three slowest Anisotropic Network Model (ANM) modes are color mapped on to the (**a**) open and (**b**) closed conformations of RBP. Two structures have the similar motion status in the three slowest ANM modes. The longitudinal and transverse axes are shown with the dashed black lines in the figures, which represents the hinge rotation axis. Mode 1 is a rotation motion of the two domains around the hinge rotation axis. In the mode 2, the two domains open and close with respect to each other. In the mode 3, the two domains make a twisting motion. The arrows indicate the direction of fluctuations. The figures were generated using the program PYMOL.

The lowest frequency ANM modes of RBP exhibit the characteristics of concerted motions that coincide strikingly well with the experimental findings. In Figure 5, Mean-square fluctuations of the three slowest ANM modes are displayed with a color-coded ribbon presentation of the open (A) and closed (B) RBP conformations. The longitudinal and transverse axes are shown with the dashed black lines in the figures. These represent the hinge rotation axes. The similar motion status has been found in the three slowest modes of both the ligand-free and ligand-bound form. The first ANM mode of the two domains drives a continuous rotation of one domain with respect to the other around an approximately fixed axis of rotation. This motion might be important for the molecule to adjust itself to allow all the invariant residues, which are located in the linker and on the cleft-facing surfaces of the two domains, to move closer to interact with the ligand. The second slowest model drives a hinge-binding, cleft-opening/-closing movement. This motion might be responsible for binding or release the ligand, ribose. These results are consistent with the previous study by Bjorkman and Mowbray [9]. They have described the opening motion of RBP in terms of the axis of rotation (mode 1) and angle of rotation (mode 2) necessary to align two corresponding domains of the closed and open conformations, oriented in such a way that the two other domains are superimposed [45]. In the third mode, the two domains make a twisting motion with respect to each other. As the one domain moves left/right, the other domain moves right/left as show in Figure 5 mode 3. It is postulated that ligand-bound protein initially interacts with the membrane-bound permease complex. The opening of the binding protein coupled with ligand transfer to the membrane components [1,2]. The superposition of the rotating, bending and twisting motions results in a conformation, as shown in Figure 5a, which constitutes a transition stage toward the closed conformation of RBP. The similar motion status (rotating, bending and twisting) has been found in the three modes of the ligand- bound form. It is noticeable that, the first mode of ligand binding form drives opposite rotating direction of the two domains compared with the open form, resulting in two structures. The result indicates that the allosteric transition between the open and closed conformations is induced by the association and disassociation of ligand, respectively.

### 2.3. Cross-Correlations between Residue Fluctuations

In the process of functional movement of protein, some regions in the protein have high robustness, which participate in the motion as a whole. The difference distance of all the residues between the two conformations [46] can be expressed as

(1) $$\Delta R_{ij}=R_{ij}(A)-R_{ij}(B)$$

where *R_ij_*(*A*) and *R_ij_*(*B*), indices the distance all pairs of *C_α_* atoms in the conformation A and conformation B, respectively. The results can be displayed in a two-dimensional plot, which give a good indication of rigid regions that have smaller motion in the protein.

**Figure 6:**
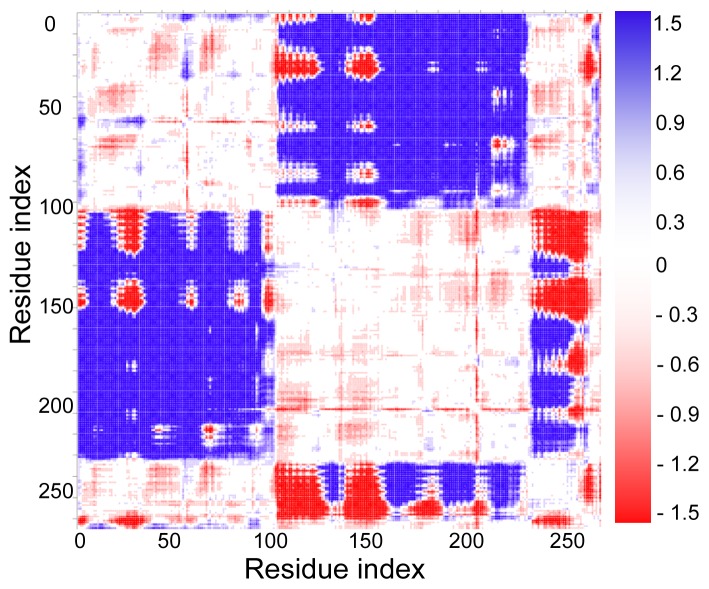
The difference-distance plot of the protein. The color changes from blue to red gradually correspond to the decrease of the distance change, as shown in the color bar on the right of the map. Both X-axis and Y-axis of the map are residue indices.

From the open-apo and closed-ligand crystal structures, the difference-distance can be calculated by Equation (1) for all *C_α_* atoms in the residues to identify the rigid region in the protein movement and the plot is shown in Figure 6. The blue and red regions mean that the change of distance between two residues is equal to or lager than 1.5 Å and −1.5 Å, respectively. The white region means that there is no distance change in the two structures, as shown in the color bar on the right of the map. Therefore, the blue and red regions have shown the relatively large changes of distance between corresponding residues. It can be seen from Figure 6, the residual distance changes within the N-(C-) domains are relatively small and this demonstrates that the N- and C-domains will keep as rigid regions during the open-closed transition, with high robustness. However, during the open-closed conformation transition, the residual distances between two domains change greatly.

**Figure 7:**
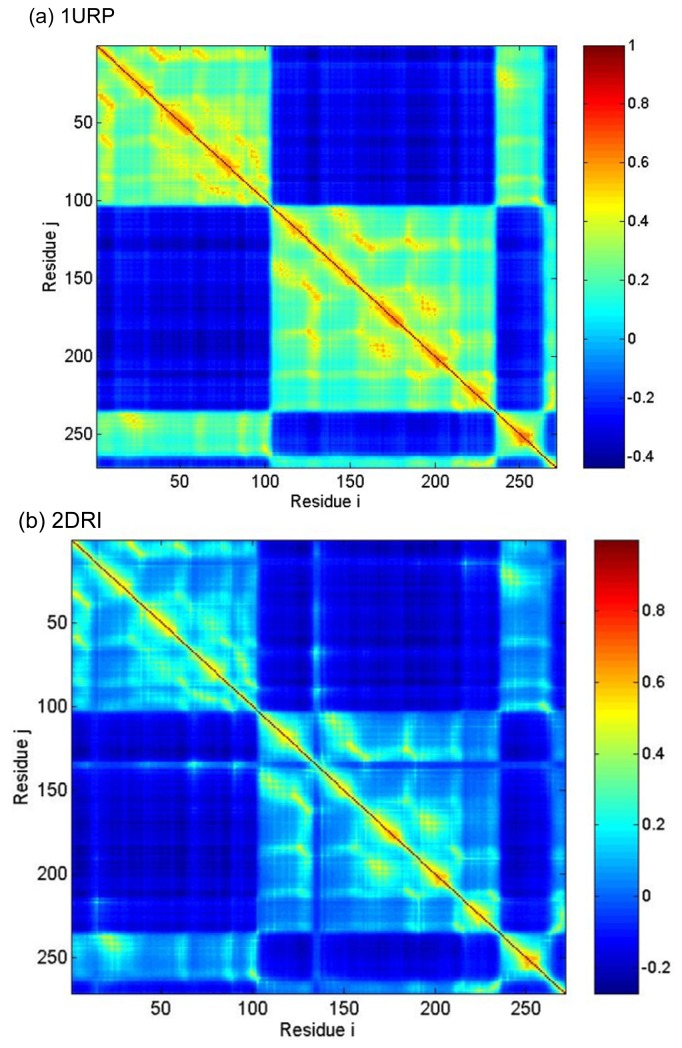
Cross-correlations map between residue fluctuations (**a**) 1URP and (**b**) 2DRI: The two axes refer to residue indices, and the colors indicate different strengths and types of interresidue orientational correlation functions [Equation (6)]. Blue and red regions correspond to negatively (opposite direction) and positively (same direction) correlated motions, respectively.

To analyze the intrinsic coupling among various residues between the N- and C-domains or among the subdomains, Figure 7 displays the interresidue cross correlation of motions in all GNM modes is calculated by Equation (6). The two axes refer to residue indices. The positive and negative limits of *C*(*i*,*j*) are 1 and −1, and correspond to pairs of residues exhibiting fully correlated (same direction, same sense) and fully anticorrelated (same direction, opposite sense) motions, respectively. Zero correlation refers to uncorrelated, or orthogonal motions. The colors in the map indicate different strengths and types of inter-residue orientational correlation functions. Blue and red regions correspond to negatively (opposite direction) and positively (same direction) correlated motions, respectively.

From Figure 7, the long-range interactions between N- and C-domains are mainly characterized by a strong and negative correlation. This is more so in the open conformation, whereas added contact points between the N- and C-domains in the closed conformation moderately reduce the negative correlation between the two domains. It is revealed that the conformational change can be described as a rigid rotation of the two domains with respect to each other, whereas the internal structure of the two domains remains largely intact. The Residues 132–136 near the exterior edge of the ligand binding cleft have a negative correlation with N-domain, while their motions are coupled with the residues near Ser68 and Gln91. The coupling provides the functionally important dynamical contact between the two domains across the cleft. Figures 6,7 shows very high consistency and it indicated that the result obtained from GNM method is consistent with the result obtained from comparing crystal structure.

### 2.4. The Fast Mode of the Motion

**Figure 8:**
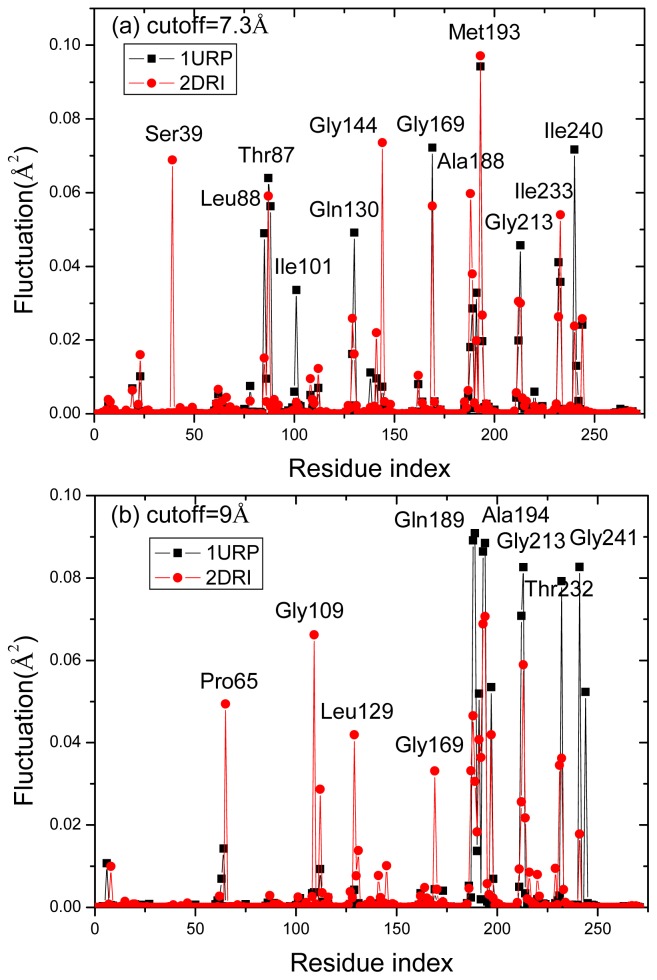
The mode shapes as averages of the fastest 10 modes of the two structures for two cases: *r_cutoff_* = 7.3 Å (**a**) and *r_cutoff_* = 9 Å (**b**).

Although the high frequency end of the spectrum is usually viewed as “uninteresting” in normal mode analysis, the peaks in the fastest GNM modes identify the residues that maintain structural integrity by resisting conformational motion [47]. Previous studies have shown that these residues correlate with experimentally determined folding nuclei or kinetically hot residue [47–49]. Figure 8 plots the mode shapes as averages of the fastest 10 modes of the two structures for two cases: *r_cutoff_* = 7.3 Å (a) and *r_cutoff_* = 9 Å (b). The results are influenced by the range of interactions considered in the analysis because the high frequency modes are highly localized. Increasing cutoff from 7.3 to 9 Å better captures the cooperative interactions by identifying the residues that participate in slightly larger local clusters. In the case *r_cutoff_* = 7.3 Å, there are six peak residues Thr87, Gly169, Met193, Gly13 Ile233 and Ile240 both in the two structures. Other peaks at 39 and 144 are located in 2DRI and 101 in 1URP, respectively. In another case *r_cutoff_* = 9 Å, the common peak residues are located at Gln189, Ala194, Gly213, Thr232 and Gly241 in the two structures. The four peak residues Pro65, Gly109, Leu129 and Gly169 are located in 2DRI. All the peak residues colored red in Figure 1a,b form a contiguous cluster distributed on the surface of RBP facing the ribose-binding pocket. These residues were known to be important in chemotaxis and transport [9]. It is found from Figure 4 that the peak residues have a lower fluctuation difference in the two structures. Previous studies have shown that the conserved residues and experimental hot spots at intermolecular binding interfaces overlap residues that vibrate with high frequencies [44]. Analysis of the organization of the conserved residues and of the hot spots indicates that they are clustered within locally highly packed regions [50].

**Figure 9:**
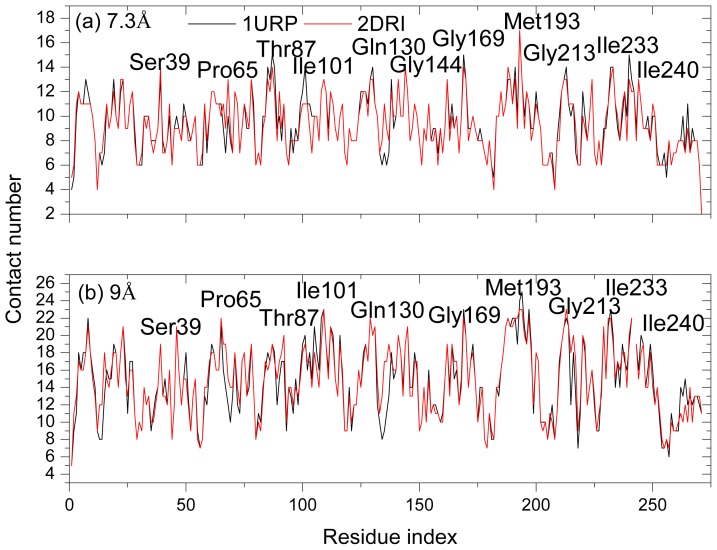
The contact number of all residues in the 1URP and 2DRI structure.

To test the result above, the conservation scores and contact number of all peak residues are calculated. The conservation scores are calculated by the ConSurf server [51]. The Expectation value (E-value) cutoff is 0.01. The conservation score is a number (called color in this website) between 1 and 9, in which 9 means the most conserved positions, 1 means the most variable positions and 5 means the average value. The results show that the conservation scores of the hot residues in the two structures are about 8.8 and it suggests that these residues are apparently conserved during evolution. Figure 7 shows the contact number of all residues in the two structures. The two structures have similar peak residues with large contact numbers. The peaks observed in Figure 8 well correspond to the peaks displayed in Figure 9 describing the kinetically hot residues. In the case *r_cutoff_* = 7.3 Å, the average contact number in 1URP and 2DRI are 14 and 13 for the hot residues, respectively, while the average contact number of all the residues in the two structures all equal to 9. In another case *r_cutoff_* = 9 Å, the average contact number in 1URP and 2DRI are all equal to 21 for the hot residues, while the average contact number of all the residues in the two structures are all equal to 15.

It indicates that the kinetically hot residues are tightly packed and have abundant contacts, which make them form the linking center of the two native structures. The site-directed mutagenesis data have showed that the surfaces involved in chemotactic and transport functions overlap extensively [52]. It has been proposed that the peak residues in the area on the cleft side of the molecule including both domains have effects on transport. A portion of the area involved in transport is also essential to chemotactic function.

## 3. Methods

The GNM has been successfully used to extract the large-scale motions in biomolecular systems with minimum computational burden [19,20,52], which is equivalent to the one additional simplifying assumption of normal mode analysis (NMA). The GNM describes a protein with a 3D elastic network of *C_α_* attached by Hookean springs where the atoms fluctuate about their mean positions. The elastic network uniforms springs of force constant γ connect the pairs of *C_α_* located within an interaction cutoff distance *r_c_*. All springs are taken to be at equilibrium for the input structure, and the input structure is assumed to be the lowest in energy, for the computed fluctuations around it. The dynamics of this network is fully defined by the *N* × *N* connectivity (cor Kirchhoff) matrix of interresidue contacts, Γ. The Kirchhoff or valency-adjacency matrix of such a structure is constructed using Equation (1).

(2) $$\Gamma =\{\begin{array}{l} -1\;\;\;if\;\;\;i\neq j\;\;\;and\;R_{ij}\leq r_{c}\\ 0\;\;\;if\;\;\;i\neq j\;\;\;and\;R_{ij}>r_{c}\\ -\sum_{i,i\neq j}\Gamma_{ij}\;\;\;if\;\;\;i=j \end{array}$$

where *i* and *j* are indices of *C_α_* and *r_c_* is the cutoff distance. Bahar and Atilgan suggested 7 Å as the reliable cutoff distance for Gaussian network model [30]. In this work, 7.3 Å is adopted to obtain a higher correlation coefficient of the B-factor between the experiment and GNM.

The inverse of this Kirchhoff matrix describes the correlations between residue fluctuations in the neighborhood of the network as shown in Equation (3), and represents the mean-square fluctuation of residue (*i* = *j*). The X-ray crystallographic B-factors, which is directly related to the mean-square fluctuations by Equation (4):

(3) $$\langle \Delta R_{i}\cdot \Delta R_{j}\rangle =(3k_{B}T/\gamma ){\left[\Gamma^{-1}\right]}_{ij}$$

(4) $$B_{i}=8\pi^{2}\langle \Delta R_{i}\cdot \Delta R_{j}\rangle /3$$

The mean-square fluctuation of the ith residue associating with the kth mode can be expressed as:

(5) $${\langle \Delta R_{i}\cdot \Delta R_{j}\rangle }_{k}=\left(3k_{B}T/\gamma \right){\lambda_{k}}^{-1}{\left[u_{k}\right]}_{i}{\left[u_{k}\right]}_{i}$$

In the GNM, the cross correlation is normalized as:

(6) $$C_{ij}=\frac{\langle \Delta R_{i}\cdot \Delta R_{j}\rangle }{{\left[\langle \Delta {R_{i}}^{2}\rangle \times \langle \Delta {R_{j}}^{2}\rangle \right]}^{1/2}}$$

GNM can only predict the relative sizes of fluctuations but not their directionalities as explained above. ANM is an analytical method that can also predicts the directionalities, and decomposes the molecular motions into a series of 3N-6 modes. In order to include the directionalities, a 3N-dimensional Hessian matrix is adopted in the ANM, instead of the N-dimensional Kirchhoff matrix in GNM, where N represents the total number of modes/residues. Atilgan *et al*. [32] gave a detailed theoretical development. For a structure with N residues, the Hessian matrix H contains *N* by *N* superelements each of size 3 by 3. The *ij*th superelement of *H* is given as:

(7) $$H_{ij}=\left(\begin{array}{ccc} \frac{\partial^{2}V}{\partial X_{i}\partial X_{j}} & \frac{\partial^{2}V}{\partial X_{i}\partial Y_{j}} & \frac{\partial^{2}V}{\partial X_{i}\partial Z_{j}}\\ \frac{\partial^{2}V}{\partial Y_{i}X_{j}} & \frac{\partial^{2}V}{\partial Y_{i}\partial Y_{j}} & \frac{\partial^{2}V}{\partial Y_{i}Z_{j}}\\ \frac{\partial^{2}V}{\partial Z_{i}\partial X_{j}} & \frac{\partial^{2}V}{\partial Z_{i}\partial Y_{j}} & \frac{\partial^{2}V}{\partial Z_{i}\partial Z_{j}} \end{array}\right)$$

where *X_i_*, *Y_i_*, and *Z_i_* are the Cartesian components of residue *i* and *V* represents the potential energy of the system. The potential between residues *i* and *j* is harmonic, modeled by a Hookean spring, for residues *i* and *j* within the cutoff distance. In the ANM, a larger cutoff distance than for GNM, calculations performed with *r_c_* varying in the range 12 ≤ *r_c_* ≤ 15 Å were found to yield almost indistinguishable results [32]. In this work, 15 Å is adopted to establish the spring connections between residues. Similarly, the inverse of *H* contains *N* by *N* superelements, with the superelements of *H*^−1^, being 3 by 3 matrices, describing the correlations between the components of Δ*R_i_* and Δ*R_j_*, specifically,

(8) $$\langle \Delta {R_{i}}^{2}\rangle =(3k_{B}T/\gamma )\cdot trace\left({\left[H^{-1}\right]}_{ij}\right)$$

The computational programs have been developed with Matlab and Results in Sections 2.1 and 2.2 have been calculated by our own program. The other GNM and ANM calculations were done using online by iGNM server [53] and ANM server [54].

## 4. Conclusions

Conformational fluctuations of the RBP have been previously studied in experimental and molecular dynamics studies [9,16,52]. In this study, we investigated the consequences of using a coarse-grained analytical method (ANM and GNM) to study the vibrational motions of RBP. This study provides information about the molecular motion of RBP and also the effect of using a coarse grained model on the motions. The results indicate that the closed-ligand structure gives a higher correlation coefficient than the open-unligand structure between the mean square fluctuations of residues evaluated, which maybe reflects the effect of the ligand ribose on the residue fluctuations of the protein. The same motion hinge axes around Ser103, Gln235 and Asp264 has been found and the open-closed conformational transition is common controlled by the two domains. The fluctuations of the ligand-binding residues in the open form have reduced remarkably comparing with the closed form, which is helpful for ligand binding or release. The superposition of the three slowest anisotropic network model modes, consisting of the rotating, bending and twisting motions of the two forms have been found, accounts for large rearrangement of domains from the ligand-free (open) to ligand-bound (closed) conformation and thus constitutes a critical component of the RBP’s functions. The kinetically “hot” residues corresponding to the high-frequency vibrating residues in the fast modes of the GNM consistent with their functions whereas the network of highly conserved residues shows almost no fluctuation difference stabilizing the two structures of RBP. The results obtained from Cross-correlation analysis and difference-distance plot indicate that the conformational change can be described as a rigid rotation of the two domains with respect to each other, whereas the internal structure of the two domains remains largely intact.

## Figures and Tables

**Table 1:** The correlation coefficient of the B-factor between the experiment and Gaussian Network Model (GNM) of RBP and five other proteins belonging to the *Escherichia coli* family.

Periplasmic binding proteins		Pdb ID	Correlation	Reference	Resolution (Å)
Leucine-binding protein	Apo	1USG	0.554	Magnusson 2004 [35]	1.53
	Complex	1USI	0.64		1.8

Nickel-binding protein	Apo	1UIU	0.492	Heddle 2003 [36]	1.85
	Complex	1UIV	0.531		1.95

Allose-binding protein	Apo	1GUD	0.69	Chaudhuri 1999 [37]	1.71
	Complex	1RPJ	0.594	Magnusson 2002 [8]	1.8

Glutamine-binding protein	Apo	1GGG	0.488	Hsiao 1996 [38]	2.3
	Complex	1ANF	0.581	Sun 1998 [39]	1.94

Maltose-binding protein	Apo	1OMP	0.653	Sharff 1992 [40]	1.8
	Complex	1ANF	0.594	Quiocho 1997 [41]	1.67

Ribose-binding protein	Apo	1URP	0.583	Bjorkman 1998 [9]	2.3
	Apo	1BA2	0.669	Bjorkman 1998 [9]	2.1
	Complex	2DRI	0.772	Bjorkman 1994 [11]	1.6
